# Supplementary material for: SyNDI: synchronous network data integration framework
Source: BMC Bioinformatics. 2018 Nov 6;19:403. doi: 10.1186/s12859-018-2426-5 (PMC6219086; doi:10.1186/s12859-018-2426-5)
Supplement: Supplementary file 5 — Files of the example presented in Additional file 4. This additional file is a zip package that contains all files used in Additional file 4. Detailed descriptions of each of these files are presented in a README file included in this zip package. (GZ 30411 kb) [file 12859_2018_2426_MOESM5_ESM.gz › SyNDITools/Meme2Fimo/meme_4.9.0_4.tar.gz!/meme_4.9.0/tests/motiph/MCM1.meme.html]

\*\*\*\*\*\*\*\*\*\*\*\*\*\*\*\*\*\*\*\*\*\*\*\*\*\*\*\*\*\*\*\*\*\*\*\*\*\*\*\*\*\*\*\*\*\*\*\*\*\*\*\*\*\*\*\*\*\*\*\*\*\*\*\*\*\*\*\*\*\*\*\*\*\*\*\*\*\*\*\*
MEME - Motif discovery tool
\*\*\*\*\*\*\*\*\*\*\*\*\*\*\*\*\*\*\*\*\*\*\*\*\*\*\*\*\*\*\*\*\*\*\*\*\*\*\*\*\*\*\*\*\*\*\*\*\*\*\*\*\*\*\*\*\*\*\*\*\*\*\*\*\*\*\*\*\*\*\*\*\*\*\*\*\*\*\*\*
MEME version 3.5.1 (Release date: 2005/12/14 00:00:05)
For further information on how to interpret these results or to get
a copy of the MEME software please access http://meme.nbcr.net.
This file may be used as input to the MAST algorithm for searching
sequence databases for matches to groups of motifs. MAST is available
for interactive use and downloading at http://meme.nbcr.net.
\*\*\*\*\*\*\*\*\*\*\*\*\*\*\*\*\*\*\*\*\*\*\*\*\*\*\*\*\*\*\*\*\*\*\*\*\*\*\*\*\*\*\*\*\*\*\*\*\*\*\*\*\*\*\*\*\*\*\*\*\*\*\*\*\*\*\*\*\*\*\*\*\*\*\*\*\*\*\*\*
\*\*\*\*\*\*\*\*\*\*\*\*\*\*\*\*\*\*\*\*\*\*\*\*\*\*\*\*\*\*\*\*\*\*\*\*\*\*\*\*\*\*\*\*\*\*\*\*\*\*\*\*\*\*\*\*\*\*\*\*\*\*\*\*\*\*\*\*\*\*\*\*\*\*\*\*\*\*\*\*
REFERENCE
\*\*\*\*\*\*\*\*\*\*\*\*\*\*\*\*\*\*\*\*\*\*\*\*\*\*\*\*\*\*\*\*\*\*\*\*\*\*\*\*\*\*\*\*\*\*\*\*\*\*\*\*\*\*\*\*\*\*\*\*\*\*\*\*\*\*\*\*\*\*\*\*\*\*\*\*\*\*\*\*
If you use this program in your research, please cite:
Timothy L. Bailey and Charles Elkan,
"Fitting a mixture model by expectation maximization to discover
motifs in biopolymers", Proceedings of the Second International
Conference on Intelligent Systems for Molecular Biology, pp. 28-36,
AAAI Press, Menlo Park, California, 1994.
\*\*\*\*\*\*\*\*\*\*\*\*\*\*\*\*\*\*\*\*\*\*\*\*\*\*\*\*\*\*\*\*\*\*\*\*\*\*\*\*\*\*\*\*\*\*\*\*\*\*\*\*\*\*\*\*\*\*\*\*\*\*\*\*\*\*\*\*\*\*\*\*\*\*\*\*\*\*\*\*
\*\*\*\*\*\*\*\*\*\*\*\*\*\*\*\*\*\*\*\*\*\*\*\*\*\*\*\*\*\*\*\*\*\*\*\*\*\*\*\*\*\*\*\*\*\*\*\*\*\*\*\*\*\*\*\*\*\*\*\*\*\*\*\*\*\*\*\*\*\*\*\*\*\*\*\*\*\*\*\*
TRAINING SET
\*\*\*\*\*\*\*\*\*\*\*\*\*\*\*\*\*\*\*\*\*\*\*\*\*\*\*\*\*\*\*\*\*\*\*\*\*\*\*\*\*\*\*\*\*\*\*\*\*\*\*\*\*\*\*\*\*\*\*\*\*\*\*\*\*\*\*\*\*\*\*\*\*\*\*\*\*\*\*\*
DATAFILE= MCM1.fasta
ALPHABET= ACGT
Sequence name Weight Length Sequence name Weight Length
------------- ------ ------ ------------- ------ ------
MCM1\_1 1.0000 18 MCM1\_2 1.0000 18
MCM1\_3 1.0000 18 MCM1\_4 1.0000 18
MCM1\_5 1.0000 18 MCM1\_6 1.0000 18
MCM1\_7 1.0000 18 MCM1\_8 1.0000 18
MCM1\_9 1.0000 18 MCM1\_10 1.0000 18
MCM1\_11 1.0000 18 MCM1\_12 1.0000 18
MCM1\_13 1.0000 18 MCM1\_14 1.0000 18
\*\*\*\*\*\*\*\*\*\*\*\*\*\*\*\*\*\*\*\*\*\*\*\*\*\*\*\*\*\*\*\*\*\*\*\*\*\*\*\*\*\*\*\*\*\*\*\*\*\*\*\*\*\*\*\*\*\*\*\*\*\*\*\*\*\*\*\*\*\*\*\*\*\*\*\*\*\*\*\*
\*\*\*\*\*\*\*\*\*\*\*\*\*\*\*\*\*\*\*\*\*\*\*\*\*\*\*\*\*\*\*\*\*\*\*\*\*\*\*\*\*\*\*\*\*\*\*\*\*\*\*\*\*\*\*\*\*\*\*\*\*\*\*\*\*\*\*\*\*\*\*\*\*\*\*\*\*\*\*\*
COMMAND LINE SUMMARY
\*\*\*\*\*\*\*\*\*\*\*\*\*\*\*\*\*\*\*\*\*\*\*\*\*\*\*\*\*\*\*\*\*\*\*\*\*\*\*\*\*\*\*\*\*\*\*\*\*\*\*\*\*\*\*\*\*\*\*\*\*\*\*\*\*\*\*\*\*\*\*\*\*\*\*\*\*\*\*\*
This information can also be useful in the event you wish to report a
problem with the MEME software.
command: meme -revcomp -dna MCM1.fasta
model: mod= zoops nmotifs= 1 evt= inf
object function= E-value of product of p-values
width: minw= 8 maxw= 18 minic= 0.00
width: wg= 11 ws= 1 endgaps= yes
nsites: minsites= 2 maxsites= 14 wnsites= 0.8
theta: prob= 1 spmap= uni spfuzz= 0.5
em: prior= dirichlet b= 0.01 maxiter= 50
distance= 1e-05
data: n= 252 N= 14
strands: + -
sample: seed= 0 seqfrac= 1
Letter frequencies in dataset:
A 0.317 C 0.183 G 0.183 T 0.317
Background letter frequencies (from dataset with add-one prior applied):
A 0.316 C 0.184 G 0.184 T 0.316
\*\*\*\*\*\*\*\*\*\*\*\*\*\*\*\*\*\*\*\*\*\*\*\*\*\*\*\*\*\*\*\*\*\*\*\*\*\*\*\*\*\*\*\*\*\*\*\*\*\*\*\*\*\*\*\*\*\*\*\*\*\*\*\*\*\*\*\*\*\*\*\*\*\*\*\*\*\*\*\*
\*\*\*\*\*\*\*\*\*\*\*\*\*\*\*\*\*\*\*\*\*\*\*\*\*\*\*\*\*\*\*\*\*\*\*\*\*\*\*\*\*\*\*\*\*\*\*\*\*\*\*\*\*\*\*\*\*\*\*\*\*\*\*\*\*\*\*\*\*\*\*\*\*\*\*\*\*\*\*\*
MOTIF 1 width = 13 sites = 14 llr = 152 E-value = 2.4e-028
\*\*\*\*\*\*\*\*\*\*\*\*\*\*\*\*\*\*\*\*\*\*\*\*\*\*\*\*\*\*\*\*\*\*\*\*\*\*\*\*\*\*\*\*\*\*\*\*\*\*\*\*\*\*\*\*\*\*\*\*\*\*\*\*\*\*\*\*\*\*\*\*\*\*\*\*\*\*\*\*
--------------------------------------------------------------------------------
Motif 1 Description
--------------------------------------------------------------------------------
Simplified A :11::3676321:
pos.-specific C :::a922::22::
probability G ::::::11:169a
matrix T a99:151244:::
bits 2.4 \* \*
2.2 \* \*
2.0 \* \*\*
1.7 \* \*\* \*\*
Information 1.5 \* \*\* \*\*
content 1.2 \*\* \*\* \*\*
(15.6 bits) 1.0 \*\*\*\*\* \*\*\*
0.7 \*\*\*\*\* \*\* \*\*\*
0.5 \*\*\*\*\* \*\*\* \*\*\*
0.2 \*\*\*\*\*\*\*\*\* \*\*\*
0.0 -------------
Multilevel TTTCCTAAATGGG
consensus ACTTAA
sequence C CC
--------------------------------------------------------------------------------
--------------------------------------------------------------------------------
Motif 1 sites sorted by position p-value
--------------------------------------------------------------------------------
Sequence name Strand Start P-value Site
------------- ------ ----- --------- -------------
MCM1\_6 + 1 1.22e-08 . TTTCCTAAACGGG CTCAA
MCM1\_3 - 4 3.18e-07 TT TTTCCAAATCGGG TCA
MCM1\_14 + 1 6.77e-07 . TTTCCTAAAGCGG GAAAT
MCM1\_11 - 4 7.73e-07 TT TTTCCTAAACAGG TTA
MCM1\_13 - 4 1.65e-06 TA TTTCCCAAAAAGG AAA
MCM1\_7 + 1 1.93e-06 . TTTCCTCTTTGGG CAAGT
MCM1\_5 - 4 1.93e-06 TT TTTCCTCTTTGGG CGG
MCM1\_10 - 4 2.64e-06 AT TTTCCCGATTGGG GTG
MCM1\_2 - 4 3.15e-06 TT TTACCAAAAAGGG CAA
MCM1\_9 - 4 5.25e-06 CT TTTCCAATAGCGG TAA
MCM1\_8 + 1 5.25e-06 . TTACCACAAAGGG TAAAA
MCM1\_4 - 4 2.35e-05 AT TTTCTTTATTGGG TTT
MCM1\_1 + 1 5.34e-05 . TATCTCAAAACGG CAAAA
MCM1\_12 - 4 6.58e-05 AA TTTCCTAGTTAAG AAC
--------------------------------------------------------------------------------
--------------------------------------------------------------------------------
Motif 1 block diagrams
--------------------------------------------------------------------------------
SEQUENCE NAME POSITION P-VALUE MOTIF DIAGRAM
------------- ---------------- -------------
MCM1\_6 1.2e-08 [+1]\_5
MCM1\_3 3.2e-07 3\_[-1]\_2
MCM1\_14 6.8e-07 [+1]\_5
MCM1\_11 7.7e-07 3\_[-1]\_2
MCM1\_13 1.7e-06 3\_[-1]\_2
MCM1\_7 1.9e-06 [+1]\_5
MCM1\_5 1.9e-06 3\_[-1]\_2
MCM1\_10 2.6e-06 3\_[-1]\_2
MCM1\_2 3.2e-06 3\_[-1]\_2
MCM1\_9 5.2e-06 3\_[-1]\_2
MCM1\_8 5.2e-06 [+1]\_5
MCM1\_4 2.4e-05 3\_[-1]\_2
MCM1\_1 5.3e-05 [+1]\_5
MCM1\_12 6.6e-05 3\_[-1]\_2
--------------------------------------------------------------------------------
--------------------------------------------------------------------------------
Motif 1 in BLOCKS format
--------------------------------------------------------------------------------
BL MOTIF 1 width=13 seqs=14
MCM1\_6 ( 1) TTTCCTAAACGGG 1
MCM1\_3 ( 4) TTTCCAAATCGGG 1
MCM1\_14 ( 1) TTTCCTAAAGCGG 1
MCM1\_11 ( 4) TTTCCTAAACAGG 1
MCM1\_13 ( 4) TTTCCCAAAAAGG 1
MCM1\_7 ( 1) TTTCCTCTTTGGG 1
MCM1\_5 ( 4) TTTCCTCTTTGGG 1
MCM1\_10 ( 4) TTTCCCGATTGGG 1
MCM1\_2 ( 4) TTACCAAAAAGGG 1
MCM1\_9 ( 4) TTTCCAATAGCGG 1
MCM1\_8 ( 1) TTACCACAAAGGG 1
MCM1\_4 ( 4) TTTCTTTATTGGG 1
MCM1\_1 ( 1) TATCTCAAAACGG 1
MCM1\_12 ( 4) TTTCCTAGTTAAG 1
//
--------------------------------------------------------------------------------
--------------------------------------------------------------------------------
Motif 1 position-specific scoring matrix
--------------------------------------------------------------------------------
log-odds matrix: alength= 4 w= 13 n= 84 bayes= 2.32193 E= 2.4e-028
-1826 -1748 -1748 166
-214 -1748 -1748 155
-114 -1748 -1748 143
-1826 244 -1748 -1826
-1826 221 -1748 -114
-14 21 -1748 66
102 21 -136 -214
117 -1748 -136 -56
85 -1748 -1748 43
-14 21 -36 17
-56 21 163 -1826
-214 -1748 233 -1826
-1826 -1748 244 -1826
--------------------------------------------------------------------------------
--------------------------------------------------------------------------------
Motif 1 position-specific probability matrix
--------------------------------------------------------------------------------
letter-probability matrix: alength= 4 w= 13 nsites= 14 E= 2.4e-028
0.000000 0.000000 0.000000 1.000000
0.071429 0.000000 0.000000 0.928571
0.142857 0.000000 0.000000 0.857143
0.000000 1.000000 0.000000 0.000000
0.000000 0.857143 0.000000 0.142857
0.285714 0.214286 0.000000 0.500000
0.642857 0.214286 0.071429 0.071429
0.714286 0.000000 0.071429 0.214286
0.571429 0.000000 0.000000 0.428571
0.285714 0.214286 0.142857 0.357143
0.214286 0.214286 0.571429 0.000000
0.071429 0.000000 0.928571 0.000000
0.000000 0.000000 1.000000 0.000000
--------------------------------------------------------------------------------
Time 0.11 secs.
\*\*\*\*\*\*\*\*\*\*\*\*\*\*\*\*\*\*\*\*\*\*\*\*\*\*\*\*\*\*\*\*\*\*\*\*\*\*\*\*\*\*\*\*\*\*\*\*\*\*\*\*\*\*\*\*\*\*\*\*\*\*\*\*\*\*\*\*\*\*\*\*\*\*\*\*\*\*\*\*
\*\*\*\*\*\*\*\*\*\*\*\*\*\*\*\*\*\*\*\*\*\*\*\*\*\*\*\*\*\*\*\*\*\*\*\*\*\*\*\*\*\*\*\*\*\*\*\*\*\*\*\*\*\*\*\*\*\*\*\*\*\*\*\*\*\*\*\*\*\*\*\*\*\*\*\*\*\*\*\*
SUMMARY OF MOTIFS
\*\*\*\*\*\*\*\*\*\*\*\*\*\*\*\*\*\*\*\*\*\*\*\*\*\*\*\*\*\*\*\*\*\*\*\*\*\*\*\*\*\*\*\*\*\*\*\*\*\*\*\*\*\*\*\*\*\*\*\*\*\*\*\*\*\*\*\*\*\*\*\*\*\*\*\*\*\*\*\*
--------------------------------------------------------------------------------
Combined block diagrams: non-overlapping sites with p-value < 0.0001
--------------------------------------------------------------------------------
SEQUENCE NAME COMBINED P-VALUE MOTIF DIAGRAM
------------- ---------------- -------------
MCM1\_1 6.41e-04 [+1(5.34e-05)]\_5
MCM1\_2 3.78e-05 3\_[-1(3.15e-06)]\_2
MCM1\_3 3.82e-06 3\_[-1(3.18e-07)]\_2
MCM1\_4 2.82e-04 3\_[-1(2.35e-05)]\_2
MCM1\_5 2.32e-05 3\_[-1(1.93e-06)]\_2
MCM1\_6 1.46e-07 [+1(1.22e-08)]\_5
MCM1\_7 2.32e-05 [+1(1.93e-06)]\_5
MCM1\_8 6.30e-05 [+1(5.25e-06)]\_5
MCM1\_9 6.30e-05 3\_[-1(5.25e-06)]\_2
MCM1\_10 3.17e-05 3\_[-1(2.64e-06)]\_2
MCM1\_11 9.28e-06 3\_[-1(7.73e-07)]\_2
MCM1\_12 7.89e-04 3\_[-1(6.58e-05)]\_2
MCM1\_13 1.98e-05 3\_[-1(1.65e-06)]\_2
MCM1\_14 8.13e-06 [+1(6.77e-07)]\_5
--------------------------------------------------------------------------------
\*\*\*\*\*\*\*\*\*\*\*\*\*\*\*\*\*\*\*\*\*\*\*\*\*\*\*\*\*\*\*\*\*\*\*\*\*\*\*\*\*\*\*\*\*\*\*\*\*\*\*\*\*\*\*\*\*\*\*\*\*\*\*\*\*\*\*\*\*\*\*\*\*\*\*\*\*\*\*\*
\*\*\*\*\*\*\*\*\*\*\*\*\*\*\*\*\*\*\*\*\*\*\*\*\*\*\*\*\*\*\*\*\*\*\*\*\*\*\*\*\*\*\*\*\*\*\*\*\*\*\*\*\*\*\*\*\*\*\*\*\*\*\*\*\*\*\*\*\*\*\*\*\*\*\*\*\*\*\*\*
Stopped because nmotifs = 1 reached.
\*\*\*\*\*\*\*\*\*\*\*\*\*\*\*\*\*\*\*\*\*\*\*\*\*\*\*\*\*\*\*\*\*\*\*\*\*\*\*\*\*\*\*\*\*\*\*\*\*\*\*\*\*\*\*\*\*\*\*\*\*\*\*\*\*\*\*\*\*\*\*\*\*\*\*\*\*\*\*\*
CPU: uracil.gs.washington.edu
\*\*\*\*\*\*\*\*\*\*\*\*\*\*\*\*\*\*\*\*\*\*\*\*\*\*\*\*\*\*\*\*\*\*\*\*\*\*\*\*\*\*\*\*\*\*\*\*\*\*\*\*\*\*\*\*\*\*\*\*\*\*\*\*\*\*\*\*\*\*\*\*\*\*\*\*\*\*\*\*
